# Privacy-Preserving Image Template Sharing Using Contrastive Learning

**DOI:** 10.3390/e24050643

**Published:** 2022-05-03

**Authors:** Shideh Rezaeifar, Slava Voloshynovskiy, Meisam Asgari Jirhandeh, Vitality Kinakh

**Affiliations:** Department of Computer Science, University of Geneva, 1227 Carouge, Switzerland; svolos@unige.ch (S.V.); meisam.asgarijirhandeh@students.unibe.ch (M.A.J.); vitality.kinakh@unige.ch (V.K.)

**Keywords:** privacy, reconstruction attack, re-identification attack

## Abstract

With the recent developments of Machine Learning as a Service (MLaaS), various privacy concerns have been raised. Having access to the user’s data, an adversary can design attacks with different objectives, namely, reconstruction or attribute inference attacks. In this paper, we propose two different training frameworks for an image classification task while preserving user data privacy against the two aforementioned attacks. In both frameworks, an encoder is trained with contrastive loss, providing a superior utility-privacy trade-off. In the reconstruction attack scenario, a supervised contrastive loss was employed to provide maximal discrimination for the targeted classification task. The encoded features are further perturbed using the obfuscator module to remove all redundant information. Moreover, the obfuscator module is jointly trained with a classifier to minimize the correlation between private feature representation and original data while retaining the model utility for the classification. For the attribute inference attack, we aim to provide a representation of data that is independent of the sensitive attribute. Therefore, the encoder is trained with supervised and private contrastive loss. Furthermore, an obfuscator module is trained in an adversarial manner to preserve the privacy of sensitive attributes while maintaining the classification performance on the target attribute. The reported results on the CelebA dataset validate the effectiveness of the proposed frameworks.

## 1. Introduction

Deep learning has been widely applied in many computer vision applications in recent years, with remarkable success. Much progress in deep learning has been made possible thanks to accessible computational power and the widely available datasets needed for training. The necessity of memory and computational power has incentivized many companies such as AMAZON, Google, and IBM to provide their customers with platforms offering Machine Learning as a Service (MLaaS). MLaaS runs on a cloud environment and covers most infrastructure issues such as data pre-processing, model training, and model evaluation. Hence, the users can deploy their machine learning models by simply uploading their data (e.g., images) into the cloud server.

With all the promises made by MLaaS, this scheme introduces various privacy challenges for both users and the service provider. From one point of view, the service providers are concerned that an adversary could be disguised as a client to steal their model parameters. On the other hand, users are worried that sensitive information might be revealed to unauthorized third parties by uploading their raw data into the cloud server [1]. Furthermore, in some financial or medical data applications, it might not be legally allowed for the user to upload and submit raw data to the cloud server. One widely used solution is to share a feature representation of data instead. However, the adversary can still exploit the privacy leakage in the feature representation and design attacks targeting various objectives.

There are mainly two types of attacks regarding the privacy of users’ data: attribute inference attack and reconstruction attack [1,2]. In the reconstruction or model inversion attack, the adversary’s goal is to reconstruct the original data given the shared feature representation. Whereas in attribute inference attack, the adversary is interested in identifying certain sensitive attributes in the data such as age, gender, race, etc.

In this paper, we consider an image classification task in which users send their original data to the cloud service provider. The adversary, a malicious user or the MLaaS provider, wishes to exploit the privacy leakage in the shared feature representation targeting reconstruction or attribute inference attack.

The rest of the paper is organized as follows: Problem formulation and assumptions are introduced in Section 2. Section 3 reviews the related work. Two defense frameworks against the reconstruction attack and attribute inference attack are proposed in Section 4 and Section 5, respectively. Finally, Section 6 concludes this work along with suggestions for future work.

## 2. Problem Formulation

**Figure 1:**
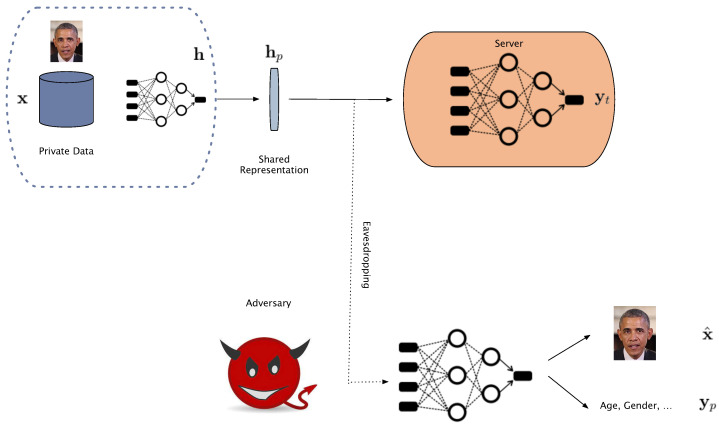
Threat model. The user sends the private representations to the server for final classification. Eavesdropping on the private features, the adversary wishes to reconstruct the original data or infer sensitive attributes. The adversary does not have access to the local obfuscation mechanism used by the user, shown in blue dashed lines.

As shown in Figure 1, given the high dimensional images in the dataset $x\in R^{n}$, users or data owners intend to share a feature representation h for the specific utility task, image classification. Let $Y_{t}$ denote the corresponding labels for the target class that the central classifier is trained to predict them and let $Y_{p}$ denote the label information for the private and sensitive attribute. Concerned about the privacy leakage in the shared representations, the users, as the defenders, apply an obfuscation mechanism on the shared features before releasing them to the public as $h_{p}$. The defender’s ultimate goal is to maintain a good classification performance while preserving their privacy.

On the other hand, having access to a collection of original images and their corresponding protected features $D=\{\left(x_{1},h_{p_{1}}\right),\left(x_{2},h_{p_{2}}\right),\cdots x_{N},h_{p_{N}})\}$, the adversary aims to reconstruct the original data or recognize sensitive attributes such as age, gender, etc. Therefore, in this setting, the utility is a classification task and privacy is defined as the attacker’s ability to reconstruct the original data or re-identify the sensitive attributes.

## 3. Related Work

Several techniques have been introduced to preserve the users’ data privacy, such as image obfuscation, homomorphic encryption, secure multi-party computation, and private feature representation.

Classical image obfuscation: In image obfuscation techniques, the original image is perturbed to hide sensitive information or details and make it visually unidentifiable. Conventional methods include pixelating [3], blurring [3,4], and masking [5]. However, as discussed in [6,7], these protected images can still be identified or reconstructed using deep learning-assisted methods. Recently, more advanced frameworks of deep obfuscation based on deep generative models have been introduced [8,9,10].

Homomorphic encryption: Homomorphic encryption (HE) is another method that allows one to carry out computations on encrypted data without the need for decryption [11]. This means that data can be processed securely even though they have been outsourced in untrusted and public environments. HE can be categorized into three types, namely partially homomorphic (PHE), somewhat homomorphic (SWHE), and fully homomorphic encryption (FHE) [11]. However, the operations in HE are limited to be represented as a polynomial of a bounded degree. They cannot, therefore, be used with complicated and nonlinear computation functions. Moreover, HE is highly computationally intensive and leads to an extremely slow training process.

Deep and private feature sharing: With the recent advancements of deep models, a new line of work has been introduced to share deep private and obfuscated feature representations of images. Osia et al. [12] considered a client-server setting in which the deep model architecture is separated into two parts: a feature extractor on the client’s side and a classifier on the cloud. The extracted features are then protected against attribute inference attacks by adding noise and Siamese fine-tuning. However, their proposed framework is not feasible during training due to its interactive training procedure and high communication throughput between the clients and servers [13].

Later, Li et al. proposed PrivyNet, a private deep learning training framework [13]. PrivyNet splits a neural network into local and cloud counterparts. The feature representations of private data are extracted using the local model while the cloud neural network is trained on publicly released features for the target classification task. The authors considered a reconstruction attack on the shared features and measured privacy through the reconstruction error. In ref. [14], the authors used an adversarial training scheme between an encoder and a classifier to preserve the privacy of intermediate encoded features from attribute inference attacks.

Along the same line of research, Lie et al. [15] introduced an adversarial privacy network called PAN to learn obfuscated features. The learned that obfuscated features are designed to be effective against both reconstruction attacks and attribute inference attacks. Similarly, DeepObfuscater was introduced in ref. [16], and the authors extended PAN to include perceptual quality.

In the context of privacy of published datasets, Huang et al. [17] proposed a framework based on a minmax game between a privatizer and an adversary. By employing generative adversarial networks (GAN) in their framework, users can directly learn to privatize their dataset without having access to the dataset statistics.

## 4. Defense against a Reconstruction Attack

This section introduces a framework to maintain a good classification accuracy while avoiding the invertibility of shared representations. In other words, the proposed framework is designed to keep only relevant information for the specific classification task. The model consists of three modules: encoder, obfuscator, and classifier. The encoder is trained using supervised contrastive loss to provide maximal discrimination for the classification task. The encoded features are obfuscated by minimizing their statistical correlation to the original input images. Finally, a classifier is jointly trained to maintain the classification performance.

### 4.1. Proposed Architecture

**Figure 2:**
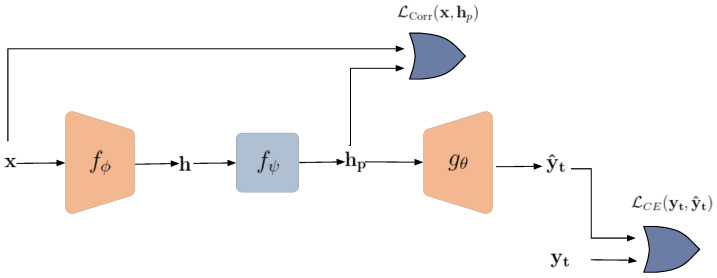
General diagram of the proposed framework for defense against reconstruction attack. $L_{CE}$ denotes cross-entropy and $L_{\mathrm{Corr}}$ stands for a similarity metric.

The overall private data-sharing framework, shown in Figure 2, consists of three steps:

1. An *encoder* $f_{\phi }$ is pre-trained on the public data using supervised contrastive loss. The encoder is later used to extract discriminative representation for the targeted classification task;
2. An *obfuscator* $f_{\psi }$ is learned to remove irrelevant information in representation h by minimizing its correlation to the original data x;
3. A *classifier* $g_{\theta }$ is jointly trained with the obfuscator to ensure that the useful information for the intended classification task is preserved in the obfuscated representation.

#### 4.1.1. Encoder

**Figure 3:**
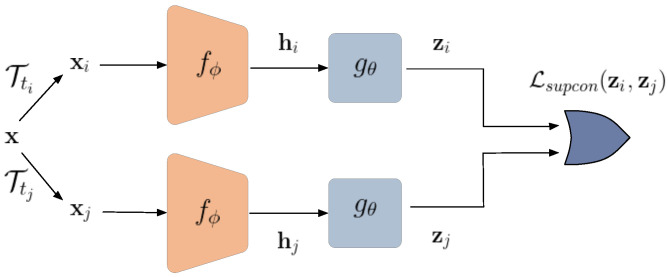
Encoder training using supervised contrastive learning.

As shown in Figure 3, the encoder $f_{\phi }$ is initially trained with a contrastive loss to output a well-discriminated feature representation. To this end, we used a ResNet backbone with contrastive loss similar to the SimCLR approach [18].

The basic idea behind contrastive learning is to pull similar instances denoted as positive pairs together and push dissimilar ones, negative samples, apart. Given a random augmentation transform $T_{t}\left(.\right)$, two different views $x_{i},x_{j}$ of the same image x are considered as positive pairs, and the rest of the batch samples as negative pairs. A projection head $g_{\theta }\left(.\right)$ maps the feature representations of the base encoder to the latent embedding z [18]:

(1) $$\begin{array}{cc} \; & x_{i}=T_{t_{i}}\left(x\right),\;h_{i}=f_{\phi }\left(x_{i}\right),\;z_{i}=g_{\theta }\left(h_{i}\right);\\ \; & x_{j}=T_{t_{j}}\left(x\right),\;h_{j}=f_{\phi }\left(x_{j}\right),\;z_{j}=g_{\theta }\left(h_{j}\right). \end{array}$$

Using cosine similarity, the similarities between positive pairs are maximized while the negative ones are minimized. The self-supervised contrastive loss is defined as:

(2) $$L_{ssl}=-\sum_{i}\log\frac{\exp(\mathrm{sim}(z_{i},z_{j}))}{\sum_{k,k\neq i}\exp\left(\mathrm{sim}(z_{i},z_{k})\right)}.$$

This idea was further extended to include target class information in the loss where feature representations from the same class are pulled closer together than those from different classes [19].

(3) $$L_{supcon}=-\sum_{i}\frac{1}{\left|P(i)\right|}\sum_{p\in P(i)}\log\frac{\exp(\mathrm{sim}(z_{i},z_{p}))}{\sum_{k,k\neq i}\exp\left(\mathrm{sim}(z_{i},z_{k})\right)},$$

where P(i) are all the positive samples belonging to the same class as $x_{i}$.

#### 4.1.2. Obfuscator

The obfuscator $f_{\psi }$ is trained to avoid the invertibility of shared feature representation. From an information-theoretic point of view, $X\to H\to \hat{X}$ forms a Markov chain. To mitigate the reconstruction attack, $I(X,\hat{X})$ should be minimized. A widely used approach is to jointly train an adversary image decoder to achieve reconstruction disparity by minimizing the Structural Similarity Index Measure (SSIM) [20]. This is done through a min-max optimization game between the obfuscator and adversary decoder.

Nevertheless, considering the information processing inequality based on the above Markov chain, minimizing the mutual information between the original image X and the feature representation H upper bounds the $I(X,\hat{X})$ as $I\left(X,H\right)\geq I\left(X,\hat{X}\right)$.

To minimize I(X,H), one should estimate the mutual information, which is a well-known and challenging problem and would involve a more complicated optimization. To solve this issue and to accelerate and simplify the training, we adopted two statistical correlation measures between random variables, namely, Hilbert–Schmidt Independence Criterion (HSIC) [21,22] and Distance Correlation (DistCorr) [23]. Consequently, the obfuscator network $f_{\psi }$ is trained to minimize the correlation between the original images and the protected representation:

(4) $$L_{\mathrm{Corr}}=\mathrm{Corr}\left(x,h_{p}\right),$$

where Corr(.) can be either based on distance correlation DistCorr or Hilbert–Schmidt Independence Criterion HSIC. The idea of minimizing the statistical dependencies of features has been around in the literature of federated or distributed learning and physics [24,25,26].

Hilbert–Schmidt Independence Criterion (HSIC): Let F be a reproducing kernel Hilbert space (RKHS), with the continuous feature mapping ϕ(x) and kernel function $k\left(x,x^{\prime }\right)=\langle \phi \left(x\right),\phi \left(x^{\prime }\right)\rangle$. Similarly, assume G be an RKHS, with the continuous feature mapping ψ(h) and kernel function $k\left(h,h^{\prime }\right)=\langle \psi \left(h\right),\psi \left(h^{\prime }\right)\rangle$.

The cross-covariance operator $C_{xh}:G\to F$ can be defined as [21,22]:

(5) $$C_{xh}:=E_{p(x,h)}[\left(\phi \left(x\right)-\mu_{x}\right)⊗\left(\psi \left(h\right)-\mu_{h}\right],$$

where ⊗ is the matrix product and $\mu_{x}=E_{p(x)}\left[\phi \left(x\right)\right]$, $\mu_{h}=E_{p(h)}\left[\psi \left(h\right)\right]$. The largest singular value of the cross-covariance operator $∥C_{\mathrm{xh}}∥$ is zero if and only if x and h are independent

The Hilbert–Schmidt Independence Criterion is defined as the squared Hilbert–Schmidt norm of the associated cross-covariance operator $C_{\mathrm{xh}}$:

(6) $${\mathrm{HSIC}}_{x,h}\left(F,G\right)={∥C_{\mathrm{xh}}∥}_{HS}^{2}.$$

Distance Correlation (DistCorr): Let X and H be two random vectors with finite second moments. Assume that (X,H), $\left(X^{\prime },H^{\prime }\right)$, $\left(X^{\prime \prime },H^{\prime \prime }\right)$ are independent and identically distributed. Then, the distance covariance can be defined as:

(7) $$\begin{array}{cc} \mathrm{dCov}(X,H)= & E(|X-X^{\prime }||H-H^{\prime }|)\\ \; & +E(|X-X^{\prime }|)E(|H-H^{\prime }\left|\right)\\ \; & -2E(|X-X^{\prime }||H-H^{\prime \prime }|), \end{array}$$

where |.| is the pairwise distance. Subsequently, the definition of the distance correlation will be:

(8) $$\mathrm{DistCorr}\left(X,H\right)=\frac{\mathrm{dCov}(X,H)}{\sqrt{\mathrm{dCov}(X,X)\mathrm{dCov}(H,H)}}.$$

#### 4.1.3. Classifier

The classifier $g_{\theta }$ is a lightweight neural network with two fully connected layers and Relu activation functions. The classifier is jointly trained with the obfuscator to maintain the classification accuracy for the utility task:

(9) $$\left(\hat{\theta },\hat{\psi }\right)={\mathrm{argmin}}_{\theta ,\psi }L_{CE}\left(y_{t},{\hat{y}}_{t}\right)+\gamma L_{\mathrm{Corr}}\left(x,h_{p}\right),$$

where γ is the utility-privacy trade-off parameter. $L_{CE}$ denotes the cross-entropy between the utility attribute $y_{t}$ and its estimate ${\hat{y}}_{t}$ and $L_{\mathrm{Corr}}$ denotes either DistCorr or HSIC according to Equations (6) and (8).

### 4.2. Experimental Results

#### 4.2.1. Experimental Setup

Dataset: We conducted experiments on a celebrity face image dataset, CelebA [27], which consists of over 20,000 celebrity images, where each image is annotated with 40 attributes. Every input image is center-cropped by 178×178 and then resized to 128×128. We select the “gender” attribute for our intended classification task.

Attacker setup: The adversary has a set of publicly available protected representations $h_{p}$ with the corresponding original images x and aims to train a decoder to reconstruct the original image for the model inversion attack.

#### 4.2.2. Visualizations of Encoded Features

**Figure 4:**
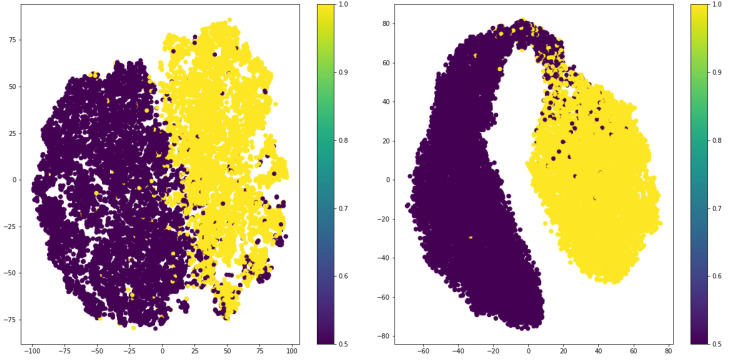
T-SNE visualization of output features for unsupervised and supervised contrastive losses.

This section investigates the effect of using supervised contrastive loss in the encoded features. To do so, we visualized the 2D t-SNEs of extracted features for the target class label of “gender,” as depicted in Figure 4. As expected, the output features of the encoder trained with supervised loss are more discriminative compared to those trained in the unsupervised way.

#### 4.2.3. Classification Performance

In this section, the utility-privacy trade-off is investigated in the form of classification accuracy vs. decorrelation. More specifically, we are interested in analyzing the extent to which classification accuracy decreases if we decorrelate the features from original images. As shown in Table 1, with only 0.2 loss in the accuracy, the correlation between input images and the features drops for both similarity measures. In the case of HSIC, however, the reduction in correlation is remarkable. The considerably smaller loss in the accuracy is mainly due to the supervised contrastive loss used in training the encoder, as we obtain discriminative features with respect to the target class. In Section 4.2.4, we demonstrate that an attacker can still reconstruct completely recognizable images using these discriminative features. Consequently, the obfuscator aims at removing all the redundant information about the images and only keeping the ones related to the intended classification task.

#### 4.2.4. Reconstruction Attack

**Figure 5:**
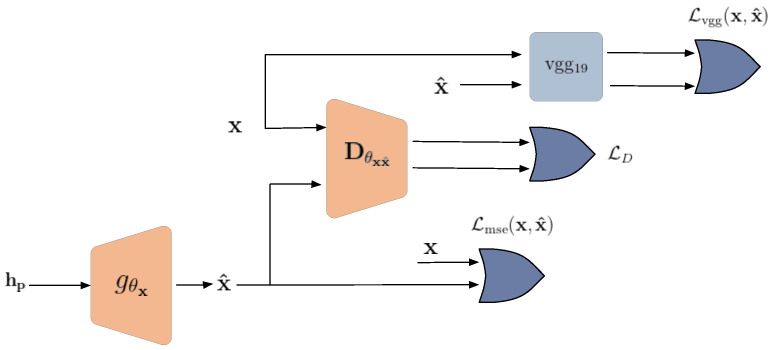
Adversary model for reconstruction attack.

According to Figure 5, the adversary model for the reconstruction attack consists of a generator $G_{\theta_{x}}$ and a discriminator $D_{\theta_{x\hat{x}}}$. The generator network maps the protected and obfuscated feature representation $h_{p}$ to the image space, while the discriminator evaluates them. The discriminator network assigns a probability that the image is from the real data distribution rather than the generator distribution. Thus, the discriminator is trained to classify images as being from the training data or reconstructed from the generator:

(10) $$L_{D}=\log\left(D_{\theta_{x\hat{x}}}\left(x\right)\right)+\log(1-D_{\theta_{x\hat{x}}}\left(G_{\theta_{x}}\left(h_{p}\right)\right).$$

Therefore, the decoder and generator are trained in a min-max optimization problem:

(11) $$\min_{g_{x}}\max_{\theta_{x\hat{x}}}E_{p(x)}\left[\log\left(D_{\theta_{x\hat{x}}}\left(x\right)\right)\right]+E_{p(h_{p})}[\log\left(1-D_{\theta_{x\hat{x}}}\left(G_{\theta_{x}}\left(h_{p}\right)\right)\right].$$

To improve the performance of the generator, a perceptual loss similar to SRGAN [28] was also employed. The perceptual loss for the generator network consists of an adversarial loss and a content loss:

(12) $$L_{\mathrm{perceptual}}=\underset{\mathrm{content loss}\;}{\underset{︸}{L_{\mathrm{mse}}+L_{\mathrm{vgg}}}}+\underset{\mathrm{adversarial loss}\;}{\underset{︸}{L_{D_{g}}}},$$

and:

(13) $$\begin{array}{cc} L_{\mathrm{mse}} & =E_{p(x,h_{p})}∥x-G_{\theta_{x}}\left(h_{p}\right)∥,\\ L_{\mathrm{vgg}} & =E_{p(x,h_{p})}∥{\mathrm{vgg}}_{19}\left(x\right)-{\mathrm{vgg}}_{19}\left(G_{\theta_{x}}\left(h_{p}\right)\right)∥,\\ L_{D_{g}} & =E_{p(h_{p})}[-\log\left(D_{\theta_{x\hat{x}}}\left(G_{\theta_{x}}\left(h_{p}\right))\right)\right], \end{array}$$

where ${\mathrm{vgg}}_{19}(.)$ is the output of a pre-trained 19-layer VGG network [29].

We conducted experiments on the reconstruction attack for different correlation losses and different values of γ in Equation (9). The performance of the attack model is evaluated using multi-scale structural similarity (MSSIM) [30] and SSIM [20]. To better evaluate the effectiveness of the proposed obfuscation model, the reconstruction quality from the following scenarios has been considered:

- h: The feature representations of original images;
- $h_{x_{\mathrm{noisy}}}$: The raw images are perturbed by adding Gaussian noise and fed to the encoder to get the features;
- $h_{\mathrm{noisy}}$: The feature representations of original images are perturbed by adding Gaussian noise;
- $h_{p}$: The obfuscated and protected features.

The average SSIM and MSSIM for reconstructed images from the protected features and three other scenarios are reported in Table 2. As the SSIM and MSSIM scores were very close for both correlation measures and different values of γ, we only reported the one for DistCorr and γ=2 in Table 2. The results show that both similarity measures are dropped by a large margin with only a 0.2% loss in accuracy, therefore validating the effectiveness of the obfuscator.

**Figure 6:**
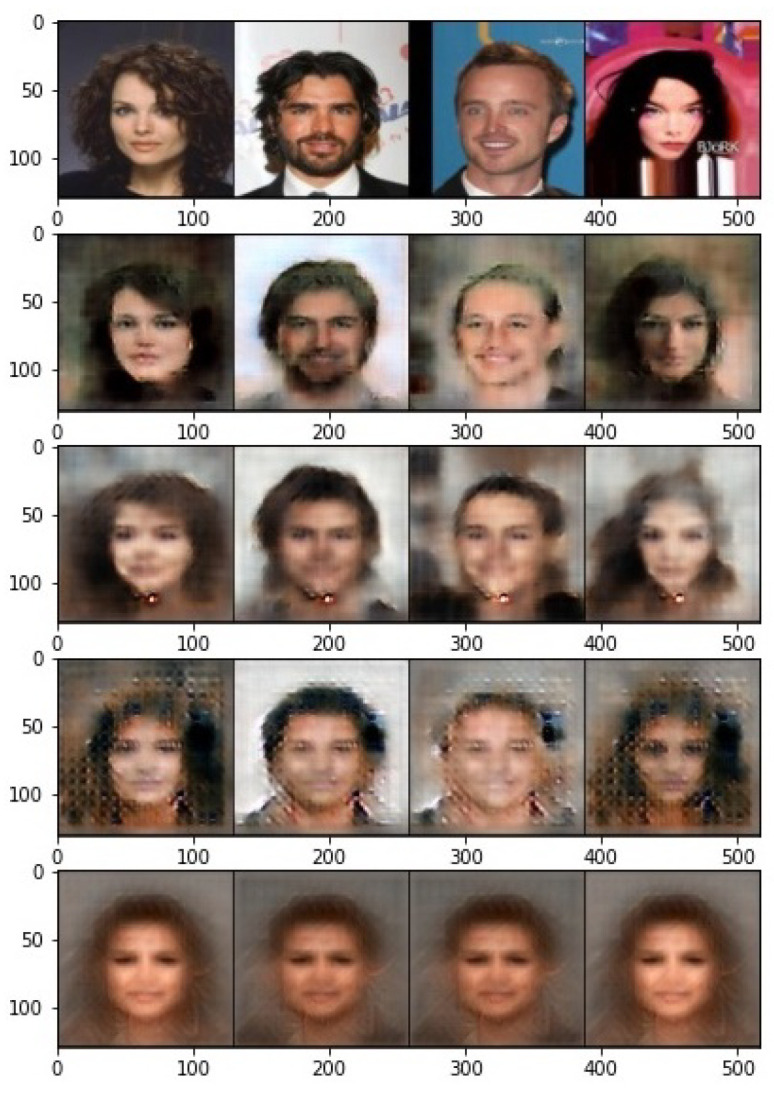
Visual performance of the reconstruction attack from different features. First row: h, second row: $h_{x_{\mathrm{noisy}}}$, third row: $h_{\mathrm{noisy}}$, and the last row: $h_{p}$ for DistCorr, γ=20.

**Figure 7:**
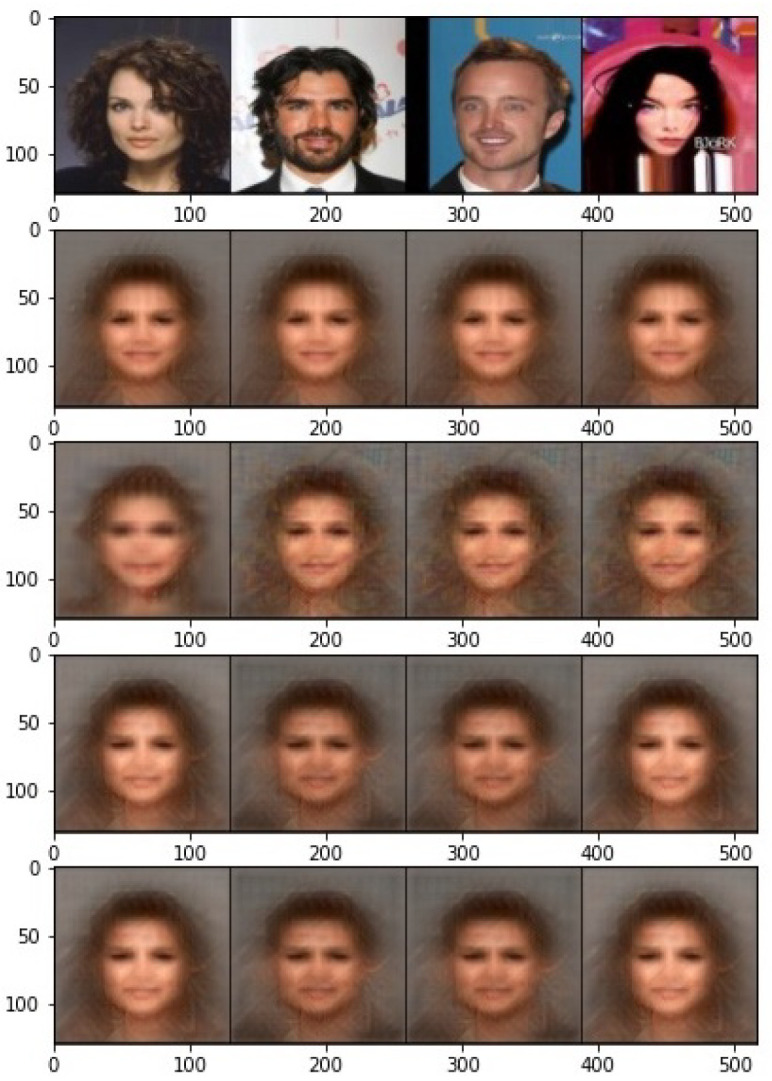
Visual performance of reconstructed output from the protected features for different correlation measures. Firs row: original images, row 2, 3: DistCorr for γ=2,20, row 4, 5: HSIC for γ=2,20.

Moreover, the visualization of the reconstructed images is illustrated in Figure 6. The reconstructed images from the raw features are completely recognizable, but not very sharp. This is mainly because the encoder is trained with the supervised contrastive loss, where the information about the target class is mostly left in the last layer. On the other hand, the output images become completely unrecognizable with our proposed obfuscator, and even a powerful decoder can only output an average image. To further investigate the effect of correlation measure and γ in Equation (9), the output images for different cases are presented in Figure 7. Even though the attacker outputs an average image for both cases of correlation measures, it is interesting to note that features learned by HSIC produce different average images for males and females. In other words, the gender information is clearly preserved in the protected representation.

## 5. Defense against an Attribute Inference Attack

Herein, our primary focus is to design a framework for defense against attribute inference attacks. The defender attempts to share a representation with relevant information about the target class label, but keeps the sensitive attribute private.

The model consists of four modules: encoder, obfuscator, target classifier, and adversary classifier. The encoder is trained using supervised and private contrastive loss to provide maximal discrimination for the classification task while protecting the private attribute. Furthermore, the encoded features are obfuscated, and the target classifier is jointly trained to maintain the classification performance. Finally, adversarial training is applied between the target classifier and the adversary classifier.

### 5.1. Proposed Architecture

**Figure 8:**
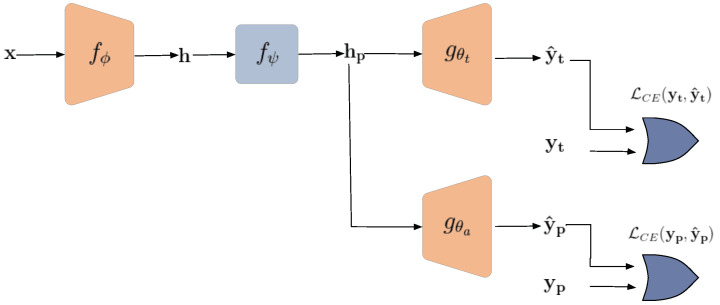
General diagram of the proposed framework for defense against an attribute inference attack.

The overall private data-sharing framework, shown in Figure 8, consists of four steps:

1. An *encoder* $f_{\phi }$ is pre-trained on the public data using supervised and private contrastive loss. The encoder is later used to extract discriminative representation for the targeted classification task;
2. An *obfuscator* $f_{\psi }$ is learned to remove relevant information in the representation h about the private attribute;
3. A *target classifier* $g_{\theta_{t}}$ is jointly trained with the obfuscator to ensure that the useful information for the intended classification task is preserved in the obfuscated representation;
4. An *adversary classifier* $g_{\theta_{a}}$ is adversely trained to minimize the classification error for the private attribute.

#### 5.1.1. Encoder

As displayed in Figure 3, the encoder $f_{\phi }$ is initially trained with supervised and private contrastive loss to output a well-discriminated feature representation and protect the private attributes. As mentioned in the previous section, the key idea behind contrastive loss is to push negative pairs apart and pull positive ones close. In a supervised contrastive loss, the positive pairs are those with the same target labels. Maximal discrimination can thus be achieved with respect to the target class.

This concept can be further extended to preserve the privacy of private attributes by allowing minimal discrimination regarding the sensitive label. In other words, for a supervised and private contrastive loss, we will assume:

- Positive pairs: Those with the same target label as the anchor image;
- Negative pairs: Those with the different target labels and the same private label as the anchor image.

Therefore, for an augmented dataset of D = {$\left(x_{1,i},x_{1,j},y_{1,t},y_{1,p}\right),\cdots \left(x_{N,i},x_{N,j},y_{N,t},y_{N,p}\right)$}, we can define the positive and negative set for each sample $x_{k}$ as:

(14) $$\begin{array}{cc} P(x_{k}) & =\{\left(x_{l,i},x_{l,j}\right)\;\mathrm{if}\;{\left(y_{k,t}=y_{l,t}\right\}}_{l=1}^{N},\\ N(x_{k}) & ={\left\{\left(x_{l,i},x_{l,j}\right)\;\mathrm{if}\;\left(y_{k,t}\neq y_{l,t}\;\&\;y_{k,p}=y_{l,p}\right)\right\}}_{l=1}^{N}. \end{array}$$

The supervised and private contrastive loss based on SupCon [19] can thus be defined as:

(15) $$L_{private-supcon}=-\sum_{i}\frac{1}{\left|P(i)\right|}\sum_{p\in P(i)}\log\frac{\exp(\mathrm{sim}(z_{i},z_{p}))}{\sum_{k\in N(i)}\exp\left(\mathrm{sim}(z_{i},z_{k})\right)},$$

where P(i) and N(i) denote positive and negative sets with respect to sample $x_{i}$. Similar to SupCon [19], Dai et al. introduced a supervised contrastive loss based on Momentum Contrast (MoCo) [31] denoted as UniCon [32]:

(16) $$L_{unicon}=\log\left(1+\sum_{\left\{k^{-}\right\}}\exp\left(s_{k^{-}}\right)\sum_{\left\{k^{+}\right\}}\exp(-s_{k^{+}})\right),$$

where *s* denotes the similarity score and $\left\{k^{-}\right\}$, $\left\{k^{+}\right\}$ are the subset of negative and positive pairs, respectively. Likewise, we can extend UniCon loss to take into account private and sensitive attributes as:

(17) $$L_{private-unicon}=\log\left(1+\sum_{k^{-}\in N\left(x_{k}\right)}\exp\left(s_{k^{-}}\right)\sum_{k^{+}\in P\left(x_{k}\right)}\exp(-s_{k^{+}}\right)).$$

#### 5.1.2. Obfuscator

The obfuscator $f_{\psi }$ is trained to hide sensitive and private attributes from the shared representation while keeping the relevant information regarding the target class label.

#### 5.1.3. Target Classifier

The classifier $g_{\theta_{t}}$ is a lightweight neural network with three fully connected layers and Relu activation functions. The classifier is jointly trained with the obfuscator to maintain the classification accuracy for the target class label:

(18) $$\left({\hat{\theta }}_{t},\hat{\psi }\right)={\mathrm{argmin}}_{\theta_{t},\psi }L_{CE}\left(y_{t},{\hat{y}}_{t}\right),$$

where $L_{CE}$ indicates the cross-entropy between the target attribute $y_{t}$ and its estimate ${\hat{y}}_{t}$.

#### 5.1.4. Adversary Classifier

The adversary classifier $g_{\theta_{a}}$ plays the role of an attacker attempting to infer private attributes using the eavesdropped features. We simulate a game between the adversary and the defender through an adversarial training procedure. The attacker tries to minimize the classification error for the private attributes as:

(19) $${\hat{\theta }}_{a}={\mathrm{argmin}}_{\theta_{a}}L_{CE}\left(y_{p},{\hat{y}}_{p}\right).$$

Meanwhile, the defender aims to degrade the performance of the adversary classifier and minimize the private attribute leakage while maintaining good performance on the target classification task. Hence:

(20) $$\hat{\psi }={\mathrm{argmin}}_{\psi }L_{CE}\left(y_{t},{\hat{y}}_{t}\right)-\gamma L_{CE}\left(y_{p},{\hat{y}}_{p}\right),$$

where γ is the utility-privacy trade-off parameter. Algorithm 1 delineates the overall steps in our proposed adversarial training procedure.
**Algorithm 1** Adversarial Training Procedure  **Input**: dataset D and parameter γ  **Output**: $\phi ,\psi ,\theta_{t},\theta_{a}$1:**for** every epoch **do**2:   Sample a minibatch from dataset3:   Train ϕ using $L_{private-supcon}$ or $L_{private-unicon}$ in Equations (15) and (16)4:**end for**5:**for** every epoch **do**6:   Sample a minibatch from dataset7:   Train ψ to minimize $L_{CE}\left(y_{t},{\hat{y}}_{t}\right)-\gamma L_{CE}\left(y_{p},{\hat{y}}_{p}\right)$8:   Train $\theta_{a}$ to minimize $L_{CE}\left(y_{p},{\hat{y}}_{p}\right)$9:   Train $\theta_{t}$ to minimize $L_{CE}\left(y_{t},{\hat{y}}_{t}\right)$10:**end for**

### 5.2. Experimental Results

This section analyzes the effectiveness of the proposed framework. For the rest of this section, we refer to utility as the classification accuracy on the target class label. Similarly, privacy is defined as the classification performance on the private and sensitive attribute.

#### 5.2.1. Experimental Setup

Dataset: We conducted experiments on a celebrity face image dataset, CelebA [27], which consists of over 20,000 celebrity images, where each image is annotated with 40 attributes. Every input image is center-cropped by 178×178 and then resized to 128×128. We select the “gender” attribute for our intended classification task and “age” with two classes of *young* and *old* as the sensitive attribute.

Attacker setup: The adversary has a set of publicly available protected representations $h_{p}$ with the corresponding original images x and their protected labels $y_{p}$ and aims to train a classifier to re-identify the protected attribute.

Defender setup: The primary goal of the defender is two-fold: the defender aims to preserve the high accuracy of classification expressed by “target accuracy” with respect to the utility attribute $y_{t}$. At the same time, the defender wishes to decrease the correct classification accuracy on the attacker’s side, which is represented by “private accuracy” with respect to the protected attribute $y_{p}$. The privacy utility trade-off is controlled by different values of γ in Equation (20). This trade-off is best achieved when, firstly, the publicly available representation $h_{p}$ is discriminative with respect to the target attribute. Secondly, there needs to be an obfuscation mechanism to remove relevant information in $h_{p}$ regarding the private attribute.

#### 5.2.2. Impact of the Obfuscator

In this section, we investigate the impact of the obfuscator. Therefore, keeping the encoder constant, we design an attribute inference attack to classify the private and sensitive attribute with and without the obfuscator. To analyze the privacy trade-off, we experimented with different values of γ in Equation (20), and the results are reported in Table 3.

As shown in Table 3, the classification accuracy significantly drops when the obfuscation is applied, thus validating the effectiveness of the obfuscator module. The obtained results show that the decline in utility is significantly small with only a 0.3–0.7% decrease in target accuracy. Moreover, the increase in γ decreases the private classification accuracy. However, in view of privacy protection, random guessing is the ultimate goal in a binary classification setting, as the adversary can flip his guess for any accuracy lower than the random guessing threshold. In order to account for this, the flipping accuracies are also reported in the last row of Table 3 accordingly. For the CelebA dataset, the class label “age” is slightly imbalanced and distributed as 75–25%; thereby, the corresponding random guessing threshold is 62.5% (0.75 × 0.75 + 0.25 × 0.25 = 0.625). Therefore, from a privacy protection point of view, the best result is obtained for γ=1 for UniCon loss.

#### 5.2.3. Privacy-Utility Trade-Off Comparison

To better evaluate the effectiveness of the proposed framework model, the privacy–utility trade-off for different scenarios has been investigated. The results in Table 3 validate the effectiveness of the obfuscator module. Putting the obfuscator aside, we are interested in analyzing the impact of using supervised and private loss compared to the conventional contrastive loss in Equation (2). To evaluate that, we considered the following scenarios:

- h: the feature representations of original images from an encoder trained with a conventional contrastive loss in Equation (2);
- $h_{x_{\mathrm{noisy}}}$: the feature representations of noisy images from an encoder trained with a conventional contrastive loss in Equation (2);
- $h_{\mathrm{noisy}}$: noisy feature representations of original images from an encoder trained with a conventional contrastive loss in Equation (2);
- $h_{\mathrm{private}-\mathrm{unicon}}$: the feature representations of original images from an encoder trained with private UniCon loss in Equation (16);
- $h_{\mathrm{private}-\mathrm{supcon}}$: the feature representations of original images from an encoder trained with private SupCon loss in Equation (15);
- $h_{p_{\mathrm{unicon}}}$: the obfuscated and protected features of the proposed framework using UniCon loss in Equation (16);
- $h_{p_{\mathrm{supcon}}}$: the obfuscated and protected features of the proposed framework using SupCon loss in Equation (15).

The privacy–utility tradeoff in the form of target and private accuracy for various settings is reported in Table 4. The final accuracies were flipped in cases lower than the random guessing threshold for a fair comparison.

**Impact of supervised and private contrastive loss**: As reported in Table 4, the accuracy on the target class is higher for both cases of SupCon and Unicon compared to the unsupervised contrastive loss. This is mainly due to the fact that there was no label information used in the conventional contrastive loss (Equation (2)). In addition, the accuracy on the private attribute is 4% lower in $h_{\mathrm{private}-\mathrm{supcon}}$ and $h_{\mathrm{private}-\mathrm{supcon}}$ compared to h, showing benefits of using supervised and private loss.

**Impact of adding noise**: Adding noise to raw images or extracted features can be considered as a defense mechanism. Injecting Gaussian noise into the data has been widely used in federated learning [33,34]. Indeed, the results in Table 4 demonstrate that the privacy increases as we add noise to the images or the features. Moreover, raising the variance of the noise leads to more privacy gain. However, the private classification accuracies for noisy data are still far from the results we can achieve using the proposed framework. Besides, by adding noise, we also lose utility as the target accuracy drops.

**Comparison to DeepObfuscator [16]**: We carefully explored and examined other papers in state-of-the-art for a fair comparison. Unfortunately, the differences in the problem formulation make this comparison difficult and unfair in some cases. For example, several works have studied the privacy leakage of a face verification system different from the attribute classification problem formulation. In ref. [35], the authors proposed an adversarial framework for reducing gender information in the final embedding vectors used for the verification system. Hence, we can argue that even though the privacy task of attribute leakage in the embeddings is the same, the utility is defined differently, thereby making the comparison infeasible.

Moreover, several studies have investigated the same utility–privacy formulation as our proposed framework. However, they differ in their overall setting. For example, Boutet et al. [36] proposed a privacy-preserving framework against attribute inference attacks in a federated learning setting. In their experiments, the main target label is “smiling,” while the protected label is the “gender” of users.

Nevertheless, a very similar problem formulation and setting are studied in ref. [16]. Li et al. [16] exploit an adversarial game to maintain the classification performance on the public class label while preserving against an attribute-inference attack. As they have used different attributes as the target and private, we re-run their obfuscator model for our public and private attributes. The DeepObfuscator model in [16] is further adapted to only consider the attribute inference attack. The results reported in Table 4 demonstrate the superior performance of the proposed method compared to DeepObfucator.

## 6. Conclusions

This paper addressed the problem of template protection against the most commonly used attacks, namely, reconstruction and attribute inference attacks. Two defense frameworks based on contrastive learning were proposed.

For defense against the reconstruction attack, we directly minimize the correlation and dependencies of encoded features with the original data, avoiding the unnecessary complications of a min-max adversarial training. Furthermore, training an encoder with the supervised contrastive loss would minimize discrimination in the feature space and remove redundant information about the original images. Hence, there is no substantial loss in classification performance, and the proposed framework provides a better utility-privacy trade-off.

In the attribute inference attack, the adversary wishes to access the private attribute given the shared protected templates. Therefore, in the first defense step, we propose an encoder trained with the supervised and private contrastive loss. Furthermore, an obfuscator module is trained in an adversarial manner to preserve the privacy of private attributes while maintaining a good classification performance. The reported results on the CelebA dataset validate the effectiveness of the proposed framework. The future work aims at designing a framework based on contrastive loss considering both reconstruction and attribute inference attacks. Another interesting avenue of research is to investigate the performance of the proposed framework on other datasets.

## Figures and Tables

**Table 1:** Classification vs. Correlation.

Correlation Type	Accuracy	DistCorr	HSIC
without	98.48	0.714	0.62
DistCorr, γ=2	98.2	0.24	0.25
DistCorr, γ=20	98.1	0.21	0.23
HSIC, γ=2	98.23	0.32	0.026
HSIC, γ=20	98.17	0.29	0.007

**Table 2:** Image reconstruction comparison.

Obfuscation	SSIM	MSSIM	Accuracy
h	0.4	0.56	98.48
$h_{x_{\mathrm{noisy}}}$	0.36	0.50	98.41
$h_{\mathrm{noisy}}$	0.30	0.43	98.37
$h_{p}$	0.19	0.16	98.2

**Table 3:** Classification accuracy on the CelebA dataset on target and private attributes for UniCon and SupCon loss and different values of γ.

Accuracy	UniCon			Supcon			w/o obfs.
	γ=1	γ=2	γ=10	γ=1	γ=2	γ=10	
target accuracy	98.37	98.34	98.30	98.33	98.31	98.30	98.34
private accuracy	32.5	25.32	19.58	25.32	25.32	17.89	82.2
100 − private accuracy	67.5	74.68	80.42	74.68	74.68	82.11	-

**Table 4:** Privacy-Utility trade-off.

	Accuracy	
	Target Accuracy	Private Accuracy
h	98.24	86.3
$h_{x_{\mathrm{noisy}}}$	98.03	86.05
$h_{\mathrm{noisy}}$	97.61	84.5
$h_{\mathrm{private}-\mathrm{unicon}}$	98.38	82.23
$h_{\mathrm{private}-\mathrm{supcon}}$	98.34	82.2
our: $h_{p_{\mathrm{unicon}}}$ and γ=1	98.30	**67.5**
our: $h_{p_{\mathrm{supcon}}}$ and γ=1	98.30	**74.68**
DeepObfuscator [16]	97.75	76.03

## Data Availability

https://mmlab.ie.cuhk.edu.hk/projects/CelebA.html (accessed on 2 January 2022).
